# Energy Metabolism Profiling of Human Colorectal Tumours

**DOI:** 10.1111/jcmm.70462

**Published:** 2025-03-05

**Authors:** Leenu Reinsalu, Sten Miller, Giuseppe Leonardo Auditano, Marju Puurand, Rafael Moreno‐Sanchez, Emma Saavedra, Vahur Valvere, Tuuli Käämbre

**Affiliations:** ^1^ Laboratory of Chemical Biology National Institute of Chemical Physics and Biophysics Tallinn Estonia; ^2^ Department of Chemistry and Biotechnology Tallinn University of Technology Tallinn Estonia; ^3^ Laboratorio de Control Metabólico, Carrera de Biología Facultad de Estudios Superiores‐ Iztacala, UNAM Tlanepantla Estado de México Mexico; ^4^ Departamento de Bioquímica Instituto Nacional de Cardiología Ignacio Chávez México City Mexico; ^5^ Oncology and Hematology Clinic, North Estonia Medical Centre Tallinn Estonia

**Keywords:** colorectal cancer, energy metabolism, glycolysis, oxidative phosphorylation

## Abstract

Colorectal cancer (CRC) is a significant global health burden, and its early detection is crucial. Novel diagnostic and prognostic methods are required for improving patient treatment, survival and quality of life. One promising approach is the analysis and understanding of the metabolic reprogramming undergone by cancer cells. Here, by analysing the changes in transcript and protein contents, activities, pathway flux and energy metabolite ratios in post‐operative CRC tumours, in comparison to adjacent healthy tissue, the energy metabolism was characterised at the molecular and functional levels. Greater expression of glucose transporter 1 and lactate dehydrogenase A (LDH), together with increased protein content and activity of LDH in tumours, suggested a higher glycolytic capability. Hexokinase transcripts, protein and activity were similar, whereas monocarboxylate transport transcripts and protein contents were lower in tumours. The creatine kinase transcripts and the adenylate kinase protein contents were lower in tumours, suggesting a functional decrease in the CRC energy transfer pathway. Notwithstanding this, oxidative phosphorylation was fully functional and exhibited higher catalytic efficiency (*Vmax*/*Km*_ADP_) in tumours, whereas the cellular energy charge was slightly lower in tumours. Remarkably, higher OxPhos catalytic efficiency correlated with advancing CRC clinical stage. The data revealed that CRC tumours exhibit a hybrid energy metabolism phenotype where both glycolysis and oxidative phosphorylation are highly active.

**Abbreviations**
AK: adenylate kinase
AMPK: AMP‐activated protein kinase
CK: creatine kinase
CRC: colorectal cancer
EC: energy charge
GLUT1: glucose transporter 1
HK: hexokinase
LDH: lactate dehydrogenase
MCT: monocarboxylate transporter
OxPhos: Oxidative phosphorylation
RT‐qPCR: Quantitative reverse transcription polymerase chain reaction
VDAC: voltage‐dependent anion channel

## 1 Introduction

In 2020, 18.1 million people worldwide were diagnosed with cancer; among these, colorectal cancer (CRC) stands as the third most prevalent form and ranks second in cancer‐related mortality following lung cancer [1]. Only 5%–10% of CRC cases occurred due to genetic predisposition, emphasising the relevant influence of environmental factors on cancer development risks. Despite the advancements in screening strategies and technology, only about 33% of all CRC cases are diagnosed at the early localised stage, and around a fifth of all diagnosed incidences already show distant metastases upon diagnosis [2]. Considering the stark contrast in the 5‐year survival rates between localised and metastasised CRC (88% vs. 16%, respectively), there is an imperative and urgent public health need to enable earlier CRC detection. In that regard, metabolomics approaches to identify diagnostic biomarkers in blood or urine have been pursued [3, 4]; however, many of the proposed metabolites are not specific for CRC. Therefore, a comprehensive understanding of the metabolic reprogramming undergone during CRC development should be achieved in order to identify enzymes and/or metabolites that are more specific for early detection and prognosis of CRC development.

Living cells rely on a constant supply of energy, which they produce by converting food‐sourced fuels into usable cellular energy carrier metabolites (e.g., ATP and creatine‐ phosphate). In cancer cells, energy metabolism, i.e., glycolysis and oxidative phosphorylation (OxPhos), undergoes significant reprogramming to sustain the accelerated cell proliferation and growth typical of neoplastic disease [5]. The predominant energy production pathway in different cancer cell types has not been completely established. The most widespread notion, the Warburg effect, suggests that cancer cells favour glycolytic energy production even in the presence of oxygen [6]. However, there is increasing evidence demonstrating that several cancer cell types exhibit high OxPhos rates [7, 8, 9]. Furthermore, cancer cells may adopt a hybrid energy metabolic state, simultaneously depending on both glycolysis and OxPhos pathways. This dynamic adaptive capacity of cancer cells has been termed metabolic plasticity [10]. Nonetheless, most of the mechanisms underlying this plasticity remain largely unknown.

Understanding the CRC development requires a comprehensive analysis at several levels, including changes in gene expression (transcript levels) and protein levels, enzyme activities, pathway fluxes, isoform profiles and their roles in cellular function [11]. While cancer has been historically considered solely as a genetic disease, it is now evident that extensive alterations occur in several cellular subsystems involved in functions like protein expression and activities, signalling and metabolic pathways, many of which cannot be solely attributed to genetic causes. Traditionally, molecular biology and biomedical research have focused on the study of single genes, individual protein targets, single metabolites or specific signalling pathways. However, an integral approach that considers the complex interplay of these components provides a more promising direction for uncovering changes in metabolic networks, diseases and understanding the mechanisms of drug effects [11]. Furthermore, significant cancer metabolic research is conducted primarily on 2D cancer cell line cultures; however, cell metabolism is profoundly influenced by the microenvironment. For instance, the metabolic phenotype of cultured cells can be modified by the conditions in which they are grown [12]. While these studies with cancer cell lines may offer valuable insights, they fail to fully represent the cancer clinical situation. Therefore, in the present study, energy metabolism reprogramming in post‐operative tumour material obtained from CRC patients was analysed.

## 2 Methods

### 2.1 Clinical Material

Human patient post‐operative tissue material samples were provided immediately after tumour removal by surgery by the Oncology and Hematology Clinic at the North Estonian Medical Centre (NEMC, Tallinn, Estonia). Only primary and treatment‐naive tumours were examined. In addition, samples of non‐cancerous mucosa tissue located at least 5 cm away from the tumour were also provided. Samples were collected in plastic containers with Mitomedium B solution (0.5 mM EGTA, 3 mM MgCl2, 60 mM K‐lactobionate, 20 mM taurine, 3 mM KH2PO4, 110 mM sucrose, 0.5 mM dithiothreitol, 20 mM HEPES, 5 μm leupeptin, 2 mg/mL fatty acids‐free bovine serum albumin, pH 7.1), stored on ice and immediately transported to the laboratory. To ensure that the control colorectal tissue was healthy, patho‐histology examination of all samples was expertly provided by NEMC pathologists. All patients were informed about the study and their respective signed informed consent letters were obtained. Coded identity protection was applied to protect the identity of the subjects. All actions concerning human subjects and follow‐up protocols have been approved by the Tallinn Medical Research Ethics Committee (decision numbers KK557 and KK558) and are in accordance with the Helsinki Declaration and Convention of the Council of Europe on Human Rights and Biomedicine.

### 2.2 RNA Extraction

To preserve cellular RNA integrity, a fraction of approximately 40% of each tissue sample was suspended in RNALater solution (Qiagen) for transportation. Upon arrival at the laboratory, the sample was frozen in liquid nitrogen and stored at −80°C. RNA was later extracted following the protocol by Untergasser [13]. The frozen tissue samples were homogenised using the TRIzol reagent (Ambion); the RNeasy Mini Kit (Qiagen) was used for RNA isolation. RNase‐free DNase I Solution (Qiagen) was used to eliminate DNA. Finally, RNA was eluted from the spin column with 30 L of RNase‐free water, and the total concentration of RNA was determined with a BioSpec‐Nano spectrophotometer (Shimadzu). The isolated RNA was stored at −80°C.

### 2.3 cDNA Synthesis and Real‐Time Quantitative Polymerase Chain Reaction

All reagents used for cDNA synthesis and quantitative reverse transcription polymerase chain reaction (RT‐qPCR) were from Applied Biosynthesis. cDNA was synthesised from 2 g of RNA using the High‐Capacity cDNA Reverse Transcription Kit with the RNase inhibitor following the protocol provided by the manufacturer. Reverse transcription reaction incubation was performed in an Eppendorf 5332 Mastercycler thermocycler.

RT‐qPCR was performed using a LightCycler 480 II instrument (Roche). The reaction mix contained TaqMan Gene Expression Master Mix (Thermo Fisher Scientific) and the FAM‐labelled TaqMan probes: actin‐β (Hs01060665_g1), for adenylate kinase AK1 (Hs00176119_m1), AK2 (Hs01123132_g1), AK4 (Hs03405743_g1), and AK6 (Hs00360444_g1); for creatine kinase CK‐BB (Hs00176483_m1), CK‐MT1 (Hs00179727_m1) and CK‐MT2 (Hs00176502_m1); for hexokinases HK1 (Hs00175976_m1) and HK2 (Hs00606086_m1); for glucose transporter 1 GLUT1 (Hs00892681_m1); for lactate dehydrogenase LDHA (Hs03405707_g1); for MCTs MCT1 (Hs00161826_m1), MCT2 (Hs04332706_m1) and MCT4 (Hs00358829_m1); and for 5'‐AMP‐activated protein kinase catalytic subunit alpha‐1 PRKAA1 (Hs01562315_m1). Milli‐Q water was used as a no‐template control to check for extraneous nucleic acid contamination. Transcript differences were estimated by the ΔΔCt method, using the β‐actin transcript for normalisation.

### 2.4 Protein Extraction for Western Blot

To extract proteins for Western blotting, the snap‐frozen samples were first ground in liquid nitrogen using a mortar and pestle. The resulting powder was transferred into 2 mL Lysing Matrix A tubes (RotaPrep) filled with radioimmunoprecipitation assay (RIPA) buffer consisting of 50 mM Tris‐HCl pH 8.0, 150 mM NaCl, 2 mM EDTA, 1% NP‐40, 0.1% SDS and supplemented with a protease inhibitor cocktail (Roche) following the manufacturer's instructions. For homogenisation, the sample was processed three times for 10 s using the Monolyzer (RotaPrep) at maximum speed. Subsequently, the homogenates were maintained under constant agitation for 2 h at 4°C, followed by centrifugation for 20 min at 16 000 × *g* at 4°C. The resulting tissue extracts were collected, aliquoted and stored at −80°C until protein quantification, using the BCA Protein Assay Kit (Thermo Fisher Scientific) according to the manufacturer's protocol.

### 2.5 Western Blot

Proteins (30 μg) from lysates were separated by 10% sodium dodecyl sulfate polyacrylamide gel electrophoresis followed by overnight electroblotting onto Immobilon P PVDF membranes, pore size 45 μm (Merck Millipore) and blocking for 30 min with phosphate‐buffered saline containing 0.05% Tween 20 (PBS‐T) and 5% bovine serum albumin (BSA) at room temperature. Next, the blot was incubated with primary antibodies (Table 1) for 2 h at 4°C in PBS‐T with 2% BSA, washed with PBS‐T and incubated with secondary antibodies using either goat anti‐rabbit IgG (H + L)‐horse radish peroxidase (Invitrogen) diluted 1:2000 or 1:4000 or rabbit anti‐mouse IgG H&L‐HRP (Abcam) diluted 1:5000 for 1 h at room temperature.

**TABLE 1:** Antibodies used for Western blot analysis.

Protein	Catalogue number	Manufacturer	Dilution	Host species
HK1	PA5‐117986	Invitrogen	1:1000	Rabbit
HK2	PA5‐29326	Invitrogen	1:2500	Rabbit
LDHA	PA5‐27406	Invitrogen	1:2000	Rabbit
MCT1	PA5‐72957	Invitrogen	1:500	Rabbit
MCT2	Sc‐50322	Santa Cruz Biotechnology	1:500	Rabbit
MCT4	Sc‐367101	Santa Cruz Biotechnology	1:500	Mouse
AK1	Sc‐365316	Santa Cruz Biotechnology	1:500	Mouse
AK2	PA5‐28611	Invitrogen	1:500	Rabbit
AK4	PA5‐61978	Invitrogen	1:500	Rabbit
AK6	10544‐1‐AP	Proteintech	1:200	Rabbit

Chemiluminescence was detected using the Super Signal West Femto Maximum Sensitivity substrate (Thermo Fisher Scientific) and imaged with the Biospectrum multispectral imaging system. Protein levels were quantified using ImageJ software. The unspecific background signal was subtracted, and the area for each protein blot was determined. The levels of protein of interest were normalised to the total protein level obtained from Ponceau S staining signal. The relative quantity of the target in each sample was assessed by comparing the normalised target quantity in each sample to the normalised target quantity in the reference sample.

### 2.6 Cell Culture

The colorectal adenocarcinoma cell line Caco‐2 (ATCC) was cultured in 100 mm diameter Falcon Corning cell culture dishes using Dulbecco's Modification of Eagle's Medium (Corning, 10‐013‐CV), which contains 4.5 g/L glucose, 584 mg/L L‐glutamine and 110 mg/L sodium pyruvate and supplemented with 1% 100× penicillin/streptomycin solution (Capricorn Scientific) and 10% fetal bovine serum (FBS Xtra, sourced from South America, Capricorn Scientific). Cell aliquots stored in liquid nitrogen were unfrozen for cell growth. Culturing was conducted in a CO2 incubator maintained at 37°C under 95% air/5% CO2. Sub‐culturing of cells was performed every 2 days by trypsinisation using 1× trypsin‐EDTA 0.5% solution (Capricorn Scientific) in DPBS (Dulbecco's Phosphate‐Buffered Saline, without calcium and magnesium, Corning).

Subsequently, the cell suspensions were centrifuged at 125 × *g* for 5 min, and the supernatant was discarded. Then, the cell number was determined using a Bürker–Türk counting chamber; aliquots of 15–20 × 10^6^ cells were resuspended in 1 mL of DPBS in 2 mL Eppendorf tubes and centrifuged as described previously. Following centrifugation, supernatants were discarded, and cell pellets were stored at −80°C for further analysis.

### 2.7 Enzyme Activities

To extract proteins for enzyme activity determinations from intact and saponin‐treated human tissue samples (10–100 mg semidried weight) and CaCo2 cells (15–20 × 10^6^), the samples were resuspended in 1 mL of 25 mM Tris‐HCl buffer pH 7.6, with 1 mM EDTA, 5 mM dithiothreitol, 0.1%–0.3% Triton X‐100, and a 20× dilution of protease inhibitor mix (Roche) in 1.5 mL Eppendorf tubes. Then, the samples were homogenised in a Retsch MM400 ball mill homogeniser with 2 metal beads (3 mm diameter) for 2.5 min at 30 Hz. The homogenised samples were then subjected to 2–3 cycles of liquid nitrogen freezing and warm water bath thawing to further facilitate the breaking of cells and membranes for complete enzyme release. After each procedure step, strong vortexing for 1 min was applied to the cell or tissue suspensions. The samples were then centrifuged at 14,000 × *g* for 2 min at 4°C, followed by recovering the supernatant and keeping it on ice for immediate enzymatic activity assays. Total protein contents were determined with the BCA protein assay kit (Thermo Fisher Scientific) according to the manufacturer's protocol, correcting for the DTT side reaction. To make rigorous comparisons with the protein content determinations and OxPhos measurements, glycolytic enzyme activities were analysed in both intact and saponin‐treated control and tumour tissue samples. All the enzymatic assays were determined under conditions of initial velocity.

Activities of HK and LDH were determined spectrophotometrically by following the NADP^+^ reduction and NADH oxidation absorbance at 340 nm, respectively, using a spectrophotometer Cary Bio 100 (Varian).

Assay for LDH activity was carried out at 37°C in 1 mL KME buffer (120 mM KCl, 20 mM MOPS, 1 mM EGTA, pH 7.2) including 0.2 mM NADH and 10–40 μg protein sample. The reaction was started by adding 1 mM pyruvate or increasing concentrations of pyruvate.

HK assay was carried out at 37°C in 1 mL KME buffer with 0.6 mM NADP^+^, 10 mM MgSO4, 1–2 units Glc6PDH, 50–200 μg protein supernatant. 10 mM ATP was added seconds before starting the reaction to avoid unspecific ATP hydrolysis by ATPases in the biological samples. The reaction was started by adding 2 mM glucose or increasing concentrations of glucose.

For the calculations of kinetic parameters (*Vmax*, *Km*, *Vmax*/*Km*), all the enzyme activities normalised to protein content at variable substrate concentrations were fitted to the Michaelis–Menten equation by non‐linear regression analysis using SigmaPlot 14.0 (Systat Software Inc.) and a NAD(P)H extinction coefficient of 6.22 mM^−1^ cm^−1^ at 340 nm.

### 2.8 Sample Preparation for Respirometry

Upon arrival at the laboratory (within 60 min after surgery), the tissue samples were placed into pre‐cooled (4°C) medium A (3 mM KH2PO4, 20 mM taurine, 5.7 mM ATP, 15 mM phosphocreatine [PCr], 9.5 mM MgCl2, 49 mM MES, 7.23 mM K2EGTA, and 2.77 mM K2CaEGTA, pH 7.1). Blood vessels and fat were removed from the tissue samples, which were then dissected into small samples (5–15 mg wet weight). These were permeabilised in medium A containing 50 μg saponin/mL for 30 min at 4°C under 360° rotatory mixing. The permeabilised samples were then washed three times for 5 min in pre‐cooled Mitomedium B without leupeptin and kept at 4°C until use in oxygraphic analyses. This saponin treatment allows for selective plasma membrane permeabilisation, leaving cholesterol‐lacking intracellular organelle membranes rather intact [14]. Such a permeabilisation procedure was required to make freely available oxidisable substrates and ADP to the mitochondria of human tissue samples.

### 2.9 Oxygraphic Measurements

Mitochondrial respiration of permeabilised tissue samples was measured in Mitomedium B at 25°C using a high‐resolution respirometer Oxygraph‐2k (Oroboros Instruments, Innsbruck, Austria). The medium was supplemented with 5 mM glutamate, 2 mM malate and 10 mM succinate to fully activate respiratory chain complexes I and II. To determine the relationship between respiration rate and exogenous ADP, increasing concentrations of ADP were added to the medium in the oxygraphic chamber. The collected data were then plotted as rates of O2 consumption and OxPhos (the basal respiration rate attained in the absence of added ADP was subtracted from the ADP‐stimulated respiration) versus ADP concentration. From these plots, the apparent affinity of OxPhos for exogenous ADP (*Km*ADP) and maximal OxPhos rate (*Vmax*) values were calculated by non‐linear regression using the Michaelis–Menten equation. Respirometry medium aliquots were withdrawn to calculate energy charges from adenine nucleotidequantification.

To activate glycolysis flux in the samples incubated in the respirometer, 0.1 mM of ATP and 10 mM of glucose were added after glutamate, malate and succinate, followed by the addition of 1 mM of ADP. To inhibit glycolysis, 6–2020 mM of 2‐deoxyglucose and 0.5 mM of iodoacetate were added before ADP. To inhibit OxPhos, 2.5 μm of rotenone, 10 μm of antimycin‐A and 2 μg/mL of oligomycin were added before ADP.

### 2.10 Adenine Nucleotide Quantification

Two millilitres from both tumour and control tissue respirometry medium assays was collected from the oxygraph chambers after the experiments and processed immediately or kept at −80°C for no longer than 2 weeks before processing. For protein precipitation, 70% HClO4 was added to the samples to a final concentration of 0.6 M final concentration followed by centrifugation at 17,000 × *g* for 10 min at 4°C. The supernatant was collected and neutralised with 2 M KHCO3 (120–600 μL depending on sample) and spun again, after which the supernatants were either directly loaded into a UPLC apparatus for measurements or freeze‐dried and resuspended in the desired volume and solvent and kept at −20°C for up to a few weeks before analysis.

Separation and quantification of adenine nucleotides was carried out with an Agilent 1290 Infinity UPLC apparatus using a reverse‐phase column Separon SGX C18 5 μm 3 × 150 mM (Tessek, Czechia). The samples were eluted as described before [14]. The concentrations of the nucleotides in neutralised oxygraphic samples were calculated from the peak areas, accounting for all dilution factors and normalised to the wet weight of tissue. The energy charge (EC) was calculated using the formula:

$$\mathrm{EC}=\left[\mathrm{ATP}\right]+0.5\times \left[\mathrm{ADP}\right]/\left[\mathrm{ATP}\right]+\left[\mathrm{ADP}\right]+\left[\mathrm{AMP}\right]$$

### 2.11 Data Analysis

Data in text and figures are presented as mean ± standard error (SEM). All plots were made by using SigmaPlot 14.0. The results were analysed using one‐way analysis of variance (ANOVA), and *p*‐values < 0.05 were considered significant.

## 3 Results

### 3.1 Glycolysis Proteins

#### 3.1.1 Glucose Transporters

**FIGURE 1:**
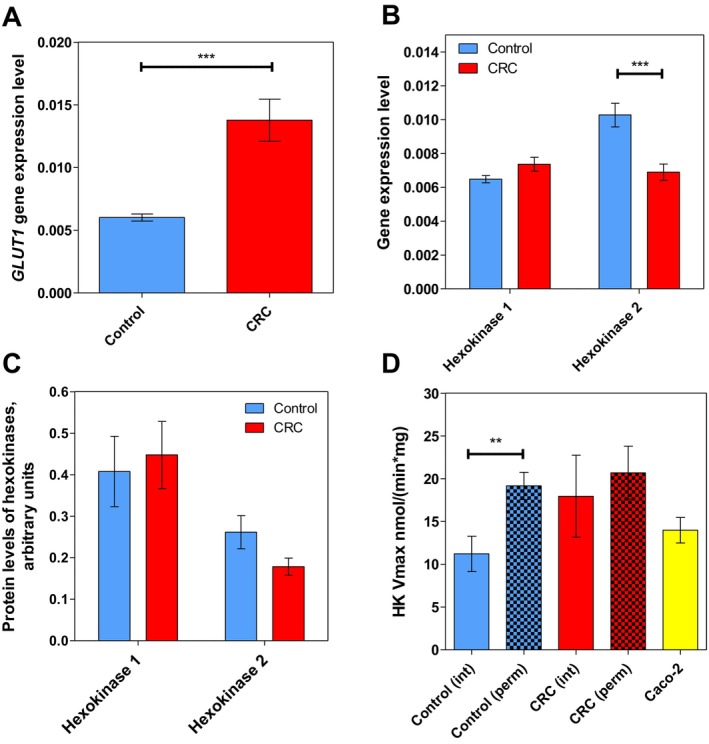
The characteristics of glucose transporter 1 (GLUT1) and hexokinases (HKs) in healthy and colorectal tumour tissue. The transcript levels of (A) *GLUT1* and (B) *HK1* and *HK2* were determined by RT‐qPCR in control colon tissue (*n* = 24) and CRC tissue (*n* = 24). (C) The protein levels of HK1 and HK2 measured by Western blot in control (*n* = 8 and *n* = 7, respectively) and CRC tissues (*n* = 8 and *n* = 4, respectively). (D) HK activity in intact control tissue (Control (int), *n* = 7), permeabilised control tissue (Control (perm), *n* = 12), intact colorectal cancer tissue (CRC (int), *n* = 7), permeabilised colorectal cancer tissue (CRC (perm), *n* = 12) and Caco‐2 cells soluble clarified cell extract (*n* = 4). **p < 0.01, ***p < 0.001 (one‐way ANOVA).

Glucose enters the cells via glucose transporters (GLUTs). Among the 14 members of the GLUT family, alterations of GLUT1 have been predominantly shown in various types of tumours [15, 16, 17]. To investigate whether glucose transport gene transcription could be affected in CRC cells, the transcript levels of the *SLC2A1* gene, which encodes GLUT1 protein, were assessed using RT‐qPCR. The results revealed a more than two‐fold increase in transcript levels in tumours compared to control colon tissue (Figure 1A), suggesting a higher content of GLUT1 protein and a heightened glucose uptake by cancer cells.

#### 3.1.2 Hexokinases

Hexokinase (HK) may couple with the voltage‐dependent anion channel (VDAC) in the outer mitochondrial membrane, thereby modulating adenine nucleotides permeability. The transcript levels of one of the main controlling steps of glycolysis, catalysed by HK1 and HK2, were evaluated (Figure 1B). While *HK1* transcript remained similar in both tissue types, *HK2* expression was 35% lower in CRC tumours. This observation supports our earlier findings showing lower *HK2* expression in CRC compared to healthy colon tissue [18, 19].

To further explore changes in HKs, their protein levels were compared. No significant differences were observed between the CRC and control tissue for each isoform (Figure 1C); however, it is noted that HK2 was detected in only four tumour samples out of nine (44%) examined, supporting the notion of HK2 downregulation in CRC. Finally, HK activity assays were carried out in both intact and saponin‐treated control and tumour tissues, as well as in Caco‐2 cells (Figure 1D). However, there were no significant HK activity differences between permeabilised control tissue and tumour intact or permeabilised tissue, and CaCo2 cells.

#### 3.1.3 Lactate Dehydrogenase

**FIGURE 2:**
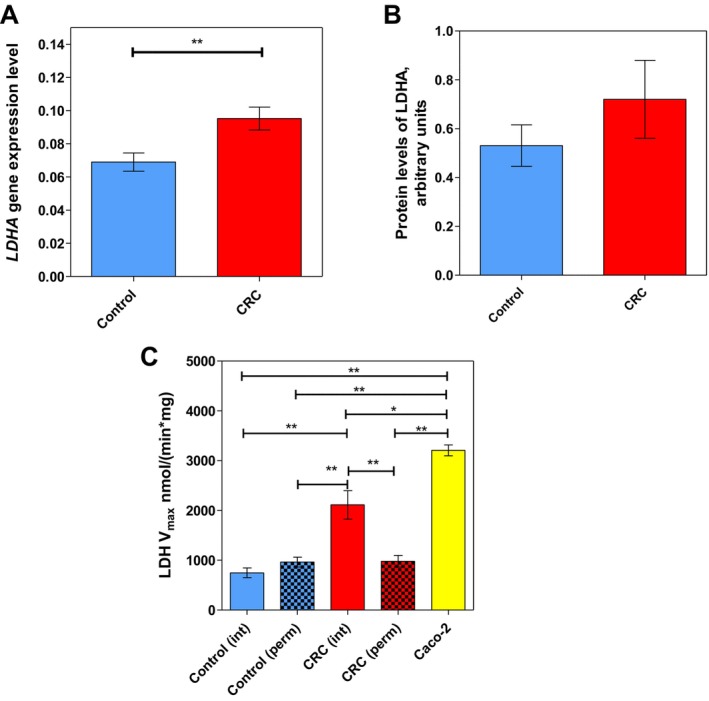
The characteristics of lactate dehydrogenase (LDH) in colorectal tumours. (A) The transcript level of *LDHA* measured by RT‐qPCR in control colon tissue (*n* = 24) and CRC tissue (*n* = 24). (B) The protein level of LDHA determined by Western blot in control colon tissue (*n* = 9) and CRC tissue (*n* = 9). (C) LDH activity in intact control tissue (Control (int), *n* = 7), permeabilised control tissue (Control (perm), *n* = 13), intact colorectal cancer tissue (CRC (int), *n* = 7), permeabilised colorectal cancer tissue (CRC (perm), *n* = 13) and Caco‐2 cells (*n* = 4). **p < 0.01, ***p < 0.001 (one‐way ANOVA).

LDHA converts pyruvate into lactate in the cytosol, thus its activity in this compartment may modulate whether pyruvate is preferentially directed towards mitochondria or transformed to lactate in the cytosol. The transcript level of *LDHA* was higher in CRC compared to control tissue (Figure 2A).

Although LDHA protein levels appeared generally higher in tumour tissue, no statistically significant difference was apparent (Figure 2B), due to considerable heterogeneity among clinical samples and a statistically insufficient sample size (*n* = 9). Notwithstanding this, LDH activity showed significant differences between different tissue types (Figure 2C). Notably, a two‐fold increase in LDH activity of intact tumour vs. intact control samples was observed.

In contrast, no difference in LDH activity between permeabilised tumour and permeabilised control samples was attained. LDH activity in Caco‐2 cells was remarkably higher, exceeding that of the CRC clinical samples by approximately 30%, with significant differences observed across all groups.

#### 3.1.4 Monocarboxylate Transporters

**FIGURE 3:**
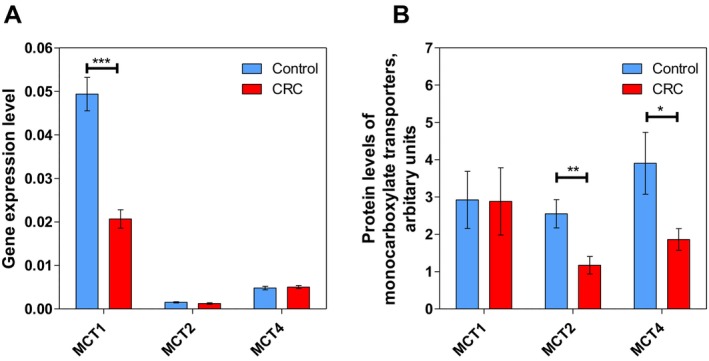
The characteristics of monocarboxylate transporters (MCTs) in colorectal tumour tissue. (A) Transcript levels (*n* = 24) and (B) protein levels (*n* = 9) of monocarboxylate transporters 1, 2 and 4 in control colon tissue and colorectal cancer tissue. *p < 0.05, **p < 0.01 and ***p < 0.001 (one‐way ANOVA).

The MCT family consists of 14 transmembrane proteins, but only MCT1, MCT2, MCT3 and MCT4 exhibit high affinity for lactate. Therefore, the transcript levels of *SLC16A1, SLC16A7* and *SLC16A3*, encoding for *MCT* 1, 2 and 4 isoforms, respectively, were analysed (Figure 3A). While the transcripts of *MCT2* and *MCT4* were similar between control and tumour tissues, the *MCT1* transcript in cancer was two‐fold lower than in control colon tissue. The transcript pattern did not agree with the protein level pattern of MCT1 and MCT4, where MCT1 protein levels were nearly identical between control and CRC tissues, whereas the protein levels of both MCT2 and MCT4 were significantly decreased in CRC (Figure 3B).

### 3.2 Energy Transfer Pathways

The energy metabolism consists of the two ATP‐producing pathways, glycolysis and OxPhos. However, besides production by these pathways, ATP must be efficiently delivered to the consumption sites. Such a required energy transfer pathway consists of the mitochondrial and cytosolic isoforms of adenylate kinase (AK) and creatine kinase (CK). To achieve a full understanding of how cancer cell energy metabolism operates and is regulated, changes in the energy transfer pathway must also be identified.

**FIGURE 4:**
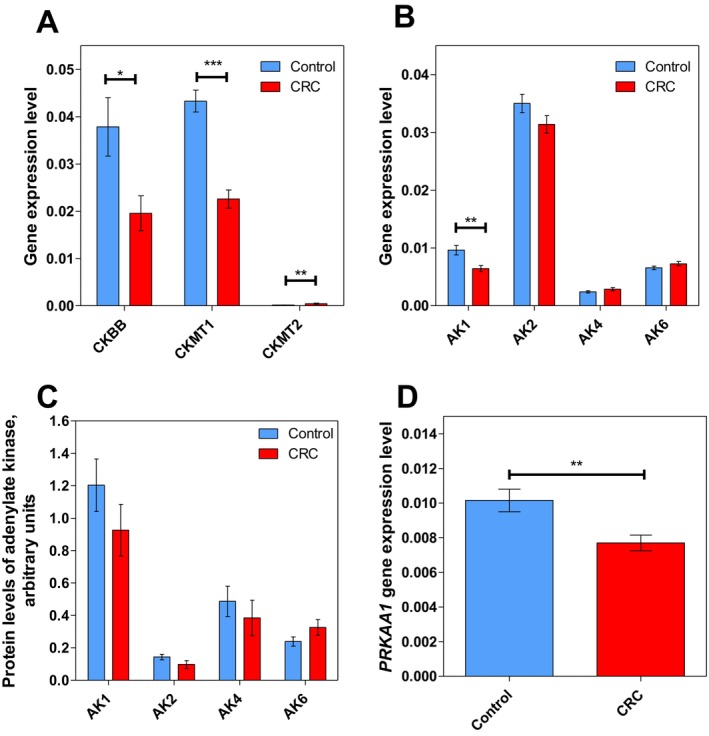
Characteristics of the energy transfer pathways in colorectal tumours. The transcript levels of (A) creatine kinase isoforms *CKBB, CKMT1* and *CKMT2* and (B) adenylate kinase isoforms *AK1*, *AK2*, *AK4* and *AK6* in control colon tissue (*n* = 24) and CRC tissue (*n* = 24). (C) The protein levels of adenylate kinase isoforms AK1, AK2, AK4 and AK6 in control colon tissue (*n* = 8) and CRC tissue (*n* = 8). (D) The transcript level of 5'‐AMP*‐activated protein kinase catalytic subunit alpha‐1* (*PRKAA1*) in control colon tissue (*n* = 24) and CRC tissue (*n* = 24). * *p* < 0.05, ** *p* < 0.01 and *** *p* < 0.001 (one‐way ANOVA).

Alterations in the CK system have been observed in many cancer types [20], including CRC. The transcript levels of three CK isoforms were determined via RT‐qPCR (Figure 4A). The results demonstrated significantly decreased levels of *CKBB* and *CKMT1* in CRC compared to control tissue. This last observation was in agreement with previous studies from our group showing that the CK activity decreases approximately two‐fold in CRC compared to normal colon tissue [19]. Interestingly, the expression of the second mitochondrial isoform *CKMT2* gene was higher in CRC tissue, although its overall expression remains low compared to other isoforms.

Downregulation of CKs could be compensated by upregulating the AK system. In this regard, it has been shown that the AK activities largely increase in CRC compared to normal colon tissue [20]. AKs catalyse the reversible transfer of phosphate groups between ADP, AMP and ATP. Nine isoforms of AKs have been identified and characterised in mammalian tissues so far [21]. Four isoforms were analysed in this study: cytosolic AK1, mitochondrial AK2 and AK4 and nucleus‐located AK6. Contrary to the hypothesis, cancer cells did not show any change in the *AK* transcripts (Figure 4B). Furthermore, while no statistically significant differences were observed in protein levels of AK isoforms (Figure 4C), there appears to be a trend of lower AK1 levels in cancer tissue, supporting the similar finding in transcript levels.

In addition to AKs, another central regulator of cellular energy homeostasis is AMP‐activated protein kinase (AMPK). AMPK is activated by low ATP and high AMP concentrations, conditions that could be caused by nutrient deprivation, hypoxia or oxidative stress. Once activated, AMPK shifts metabolism towards catabolism by phosphorylating proteins in multiple pathways [22]. Its role in cancer is still unclear. Here, the transcript level of *5*'*‐AMP‐activated protein kinase catalytic subunit alpha‐1* (*PRKAA1*) was assessed and shown to be downregulated (Figure 4D).

### 3.3 Oxidative Phosphorylation Kinetic Characteristics

**FIGURE 5:**
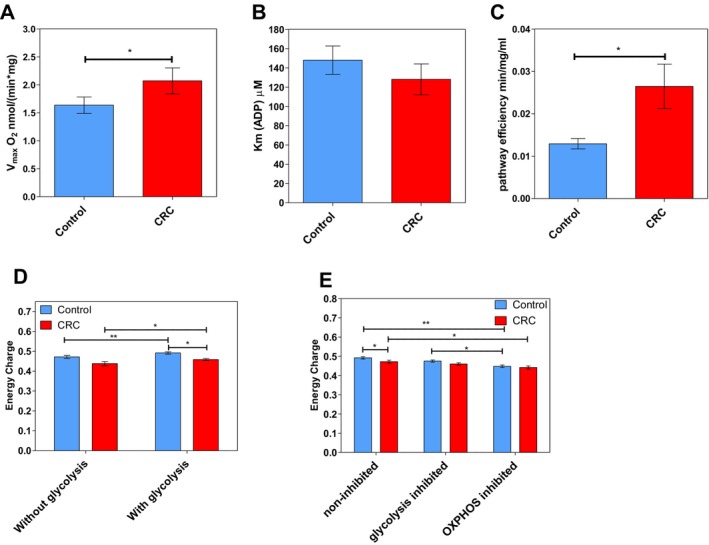
Kinetic characteristics of oxidative phosphorylation in colorectal tumours. Comparative analysis of (A) maximal ADP‐stimulated respiratory rate (*Vmax*) and (B) apparent Michaelis–Menten constant values for ADP (*Km*ADP) in control tissue (*n* = 29) and CRC tissue (*n* = 29). (C) The catalytic efficiency of the OxPhos pathway represented by the *Vmax*/*Km*ADP ratios, which were calculated from paired samples. (D) Energy charges of control and tumour tissue without (*n* = 15) and with activated glycolysis (*n* = 16), and (E) energy charges of non‐inhibited (*n* = 16), glycolysis‐inhibited (*n* = 17) and OxPhos‐inhibited (*n* = 10) states in control and tumour tissue. *p < 0.05, **p < 0.01.

Next, high‐resolution respirometry was applied to analyse the kinetics of the OxPhos flux: the values for maximal ADP‐stimulated respiration rate (*Vmax*) and the apparent Michaelis–Menten constant for exogenously added ADP (*Km*ADP) were determined (Figure 5).

A statistically significant difference in *Vmax* values between control and CRC tissue was found (Figure 5A), but no differences in *Km*ADP values (Figure 5B). The higher maximal ADP‐stimulated respiration rate in CRC suggests an increased OxPhos capacity. Further, the catalytic efficiency (*Vmax*/*KmADP* ratio) of the whole OxPhos pathway (Figure 5C) for control tissue was significantly lower (0.013 min^−1^ mg^−1^ mL) than that for CRC (0.026 min^−1^ mg^−1^ mL), indicating a more catalytically efficient system in tumour tissue.

### 3.4 Energy Charge

Analysis of the adenine nucleotide contents revealed that both control and CRC samples experienced a small increase in EC when glycolysis was active (Figure 5D,E). This suggested that reliance on glycolysis indeed affected cell energy status in healthy and cancerous tissue. In contrast, the ATP/ADP ratio (0.2–0.25 value range) showed no differences between tumour and control tissue, with or without glycolysis activated (data not shown).

However, EC in control tissues was significantly impacted by OxPhos inhibition compared to the non‐inhibited state (Figure 5E), as well as the ATP/ADP ratios (from 0.23 down to 0.17; data not shown), indicating a substantial reliance on OxPhos for maintaining energy homeostasis in non‐cancerous cells. A significant decrease was noted in the control group when comparing glycolysis inhibition to OxPhos inhibition, highlighting the distinct contributions of these metabolic pathways to cellular energy status. CRC samples also showed a significant decrease in EC (and ATP/ADP ratios from 0.25 to 0.15) with OxPhos inhibition, suggesting OxPhos‐dependent energy production in cancer cells. In contrast, CRC samples did not demonstrate a significant EC change under glycolysis‐inhibited conditions, indicating a more pronounced metabolic flexibility or a compensatory mechanism enabling energy maintenance when glycolysis is inhibited (Figure 5E).

### 3.5 OxPhos and Energy Charge Along CRC Progression

**FIGURE 6:**
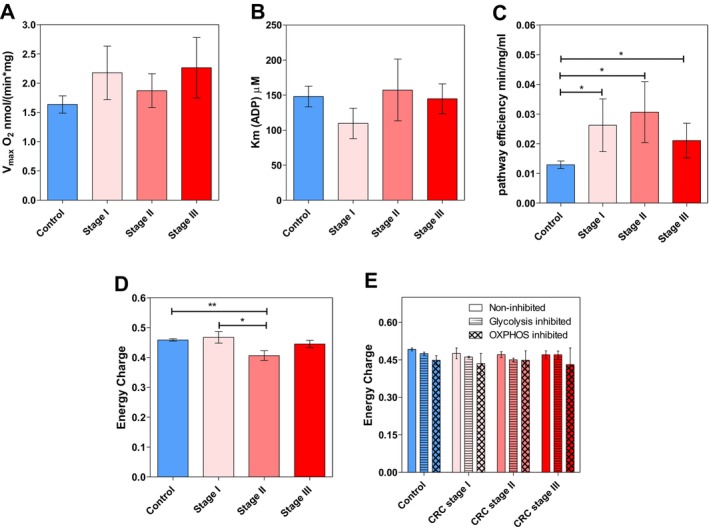
Oxidative phosphorylation and energy charge values between different colorectal cancer stages. (A) Maximal ADP‐induced respiration rate (*Vmax*) and (B) apparent Michaelis–Menten constant values for exogenously added ADP (*KmADP*) in control and tumour Stages I–III. (C) The catalytic efficiency (*Vmax/Km*) of the OxPhos pathway for control and tumour Stages I–III. (D) Energy charges of control tissue and tumour Stages I–III without glycolysis activation (*n* = 17) and (E) energy charges of tumour stages under non‐inhibited (*n* = 16), glycolysis‐inhibited (*n* = 17) and OxPhos inhibited (*n* = 10) conditions. *p < 0.05, **p < 0.01.

Searching for possible prognostic markers for aggressive cancer, OxPhos flux of CRC samples was analysed at different disease stages (Figure 6). When comparing *Vmax*, *KmADP* and EC values (and ATP/ADP ratios) across different CRC stages, no significant intergroup differences were observed, probably due to the intrinsic high sample heterogeneity among each group. Notwithstanding, a close resemblance in *Vmax* values was noted between control tissues and Stage II CRC (Figure 6A). In addition, *KmADP* values for Stage II CRC were higher than those of other stages (Figure 6B). The slight differences in these individual OxPhos kinetic parameters make the OxPhos catalytic efficiencies (*Vmax/Km*) clearly greater in the CRC stages than in the control colon tissue (Figure 6C). This suggested an enhanced OxPhos capability in CRC tumours.

Regarding EC without glycolysis activation (Figure 6D,E), control tissues showed significantly higher values compared to Stage II CRC, and there was a notable decrease between Stage I and Stage II CRC, pointing to a decrease in energy charge as the tumour progresses from Stage I to Stage II.

Finally, EC evaluations across control and CRC Stages I–III, under various metabolic conditions—non‐inhibited, glycolysis‐inhibited and OxPhos‐inhibited—revealed no significant differences between the groups (Figure 6D,E). A similar pattern of EC dependence on energy pathway was observed in control and Stage I tissues, whereas in Stages II and III, the pattern of changes seems slightly different from control.

### 3.6 OxPhos and Energy Charge Profiles of CRC Patients

Previous work with data from 32 patients showed that CRC patients who had succumbed to the disease presented significantly higher OxPhos *Vmax* values [23]. Here, additional disease progression data for all patients after 2017 were gathered, and a long‐term disease progression analysis was conducted. Among the cohort of 57 patients, a total of 12 fatalities were observed. Out of these 12 patients, 10 had initially been diagnosed with Stage 0–II CRC and 2 with Stage III–IV CRC. It is important to note that the very small number of Stage III–IV patients is because we only collected samples from patients who had not received any systemic therapy before surgery.

According to common clinical practice, patients with advanced disease mostly require some systemic therapy to achieve a resectable tumour size.

**FIGURE 7:**
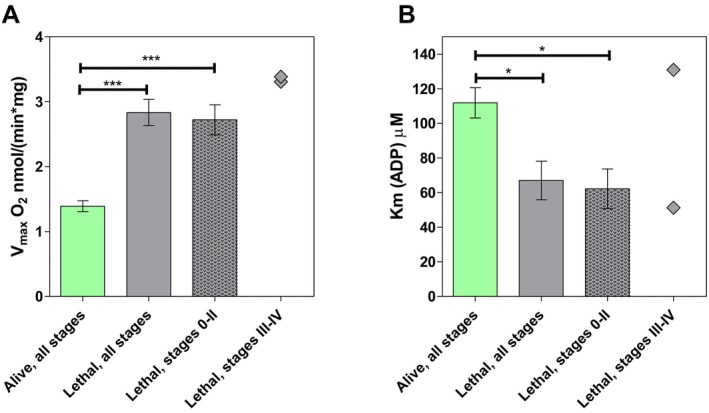
(A) Maximal ADP‐induced respiratory rate (*V*max) and (B) apparent Michaelis–Menten constant for exogenously added ADP (*KmADP*) in patients who are alive (*n* = 45, all stages including Stage IV) or have passed away (*n* = 12; Stage 0–II *n* = 10 and Stage III–IV *n* = 2) since their tumour samples were collected. *p < 0.05, ***p < 0.001.

This updated analysis showed again a significantly lower average *Vmax* value for alive patients compared to patients who had succumbed to the disease (Figure 7A). In addition, a higher average *KmADP* value was noted among the living patients (Figure 7B). The catalytic efficiency for the alive group was 0.012, whereas for the lethal groups, it was 0.042–0.043, indicating a more efficient OxPhos in the latter patients.

## 4 Discussion

### 4.1 Glycolysis

HK is one of the main rate‐controlling steps of cancer glycolysis [8, 24] and HK2 has been proposed as an independent CRC prognostic factor [25]. Coupling between VDAC and HK2 promotes the Warburg effect by channelling mitochondria‐generated ATP preferentially towards glycolysis [26]. However, co‐localisation of HK2 with VDAC has been detected in both normal colorectal mucosa and CRC [19, 27]. Thus, it is likely that the frequency and abundance of the HK–VDAC interaction depend on the cancer cell type. However, the changes observed in the present study on the transcript and protein levels of two HK isoforms in colorectal tumour and control colorectal tissue samples were not reflected in changes in total cellular HK activity. This observation suggested that the HK protein content changes were rather small or that HK1 (the most abundant HK isoform) predominated for total cellular activity over HK2.

Elevated LDH levels are often observed in the bloodstream of individuals with colon cancer [28, 29, 30]. The permeabilisation process, which presumably preserves high molecular weight proteins and mitochondria while removing cytosolic low‐molecular weight contents, resulted in a substantial decrease in LDH activity in permeabilised tumour samples compared to their intact counterparts. This observation suggested that LDH is loosely bound to intracellular components like microtubules in tumour cells. In addition, the results showed increased transcription of the *LDHA* gene together with a trend of higher LDHA protein levels and elevated LDH activity in cancer tissue, suggesting an increased lactate production capability in CRC compared to healthy colon tissue. The higher LDH activity in CaCo2 cells underscored a fundamental difference between cancer cell culture models and actual human tumours [31].

On the other hand, MCTs facilitate the transport of monocarboxylates, glycolytic intermediates like lactate, pyruvate and short‐chain carboxylic acids across the plasma cell membrane [32]. In colon tissue, the most important isoform is MCT1, which is primarily responsible for the uptake of butyrate and lactate, and MCT4, which removes intracellular lactate produced by glycolysis. The release of lactate into the tumour microenvironment contributes to an acidic pH, fostering tumour growth, invasion, and metastasis. The lactate shuttle facilitated by MCTs may serve as an energy source for metastatic cancer cells in distant organs.

Since butyrate is primarily transported into the cell via MCT1, elevated transcript levels and high protein content may partly lead colon cells to depend on short‐chain carboxylic acids supply. The higher protein content of MCT1, over that of MCT2 and MCT4, in CRC suggested an increased dependence on such short‐chain carboxylic acids supply, which may be linked to increased acetate metabolism in CRC cells [33]. The discrepancy between MCT gene and protein levels may be related to their different regulation mechanisms at the transcriptional and translational levels and cautions against over‐interpretation of their physiological meaning; hence, direct determination of activity is deemed necessary [11].

We previously demonstrated a substantial decrease in *MCT2* transcript in CRC while *MCT4* transcript was higher in CRC compared to control tissue [18]. The current study did not show a similar outcome at the level of mRNA. However, the decreased MCT2 protein level correlated with prior results. MCT2 and MCT4 have been shown to translocate to mitochondria in breast cancer cell lines [34].

### 4.2 Energy Transfer Pathways

A highly organised and efficient phosphoryl transfer system is essential to mediate intracellular communication between ATP‐consuming and ATP‐producing cellular processes for cell maintenance, growth and differentiation. During tumorigenesis, cells develop specific expression profiles of AK and CK isoforms that correlate with their oncometabolic phenotype [19, 35]. CK isoforms [19, 27, 36] catalyse the transfer of the high‐energy phosphate of ATP to creatine in the mitochondrial intramembrane space and the reverse transfer from phosphocreatine to ADP, thus synthesising ATP in the cell energy‐consuming sites and facilitating the intracellular diffusion of energy, avoiding the unspecific ADP and ATP protein binding. On the other hand, the AK family includes nine isoforms, each exhibiting distinct intracellular localisations and functional properties [21, 37, 38]. Similar to CK, the primary role of the AK system is to maintain the ATP/ADP ratio across various intracellular compartments [39]. AKs act as homeostatic metabolic regulators in both glycolysis and OxPhos, catalysing the efficient interconversion of adenine nucleotides (ATP, ADP and AMP), thereby ensuring a consistent and adequate ATP supply to fulfil cellular energy demands.

It has been demonstrated that cytosolic CK, associated with glycolytic enzymes, may support the Warburg effect by maintaining ATP homeostasis at glycolytic sites [39, 40]. However, changes in the mitochondrial outer membrane (MOM) permeability can influence the interplay between CKMTs and adenine nucleotide translocase, which catalyses the exchange of newly synthesised ATP to the cytosol by ADP entry into mitochondria. Growth of CRC is associated with upregulation of the AK system and, in parallel, with a decrease in total CK and CKMT activities [41]. Indeed, the downregulation of the CK genes in colorectal tumours was found, which was accompanied by similar AK transcripts and protein levels to those of control colorectal tissue.

Furthermore, there is a significant increase in the coupling of mitochondrial AK with OxPhos in CRC [10, 41]. Oxidative stress, accompanied by a decline in ATP levels and an ADP increase (and hence decreased ATP/ADP ratios), induces increased AK activity, leading to an elevation in intracellular AMP. In turn, AMP, acting as a secondary messenger, may activate the energy stress‐responsive AMP‐dependent protein kinase (AMPK) that stimulates ATP production through catabolic processes while inhibiting the ATP‐consuming processes involved in growth. The role of AMPK in cancer remains controversial [42, 43]. The observed slightly lower AMPK expression in colorectal tumours, whether mirrored in active protein content, suggests changes in metabolism, favouring catabolic over anabolic fluxes.

AMPK has been recognised as a tumour suppressor in certain cancers [44, 45, 46, 47] by inhibiting protein synthesis, cell proliferation and growth, since it regulates the mTORC1 pathway. However, AMPK has also been described as a contextual oncogene due to its ability to promote tumour progression, chemoresistance upon activation and cancer cell survival by maintaining NADPH homeostasis [48, 49, 50].

### 4.3 Energy Charge and ATP/ADP Ratios in CRC

In 1967, Atkinson and Walton proposed that EC derived from the adenine nucleotides contents is a fundamental metabolic thermodynamic parameter because it provides a quantitative value of the cellular energy state, reflecting the balance between energy supply and demand [51]. More recent research has delved into the intricacies of how EC reflects cancer cell metabolism and mitochondrial function, suggesting that alterations in EC can indicate disruptions in the energy balance of cancer cells, influencing their growth and survival [52].

Indeed, colorectal tumours exhibited slightly lower EC values than control tissue. EC values are established to range from 0 to 1, being close to 0 when the adenine nucleotide pool is made up of mainly AMP, 0.5 when ADP is the predominant adenine nucleotide and 1 if ATP is predominant [53]. Previous studies have documented EC values within a narrow range of 0.7–0.95, across various organisms and cell types, including liver and muscle cells. These values usually decrease under stressful conditions [52], for instance decreasing from ≥ 0.8 to less than 0.5 under nutrient depletion [54]. In contrast, our experiments consistently showed EC values ranging between 0.4 and 0.5. The estimated ATP/ADP ratio values were also lower than those determined for experiments with isolated mitochondria and intact cells and tissues.

The lower and narrower range of EC values and ATP/ADP ratios may be attributed to the use of permeabilised tissues in our experiments, which presumably retain high molecular weight enzymes that are preferentially bound to intracellular structures such as membranes, organelles and microtubules. Thus, leakage of cytosolic proteins (glycolytic enzymes) may be induced by saponin permeabilisation, thereby limiting the observable portion of glycolytic activity. In addition, tissue permeabilisation may induce increased ATPase activity. Indeed, pooling together data from saponin‐treated colorectal tumour and control tissue not subjected to lengthy incubations revealed ATP/ADP ratios of 0.3 for tumours and 0.2 for control tissues. Future research should include additional experiments using intact tumour and control clinical samples specifically for the determination of EC values, ATP/ADP ratios and glycolytic flux. Understanding such metabolic shifts could offer new avenues for cancer treatment by targeting the energy metabolism regulation mechanisms and main flux‐controlling steps [8, 11, 24, 53].

### 4.4 Kinetics of Oxidative Phosphorylation in CRC Progression and Patient Outcome

Our group has previously shown a significant difference in *Km*ADP values between glycolytic and oxidative muscle tissue [55]. The oxidative tissue shows higher *Km*ADP values, indicating a shift in the regulation of the MOM permeability by VDAC. In addition, we have also demonstrated that while control colon tissue and CRC tend to have similar *Km*ADP values, which are in agreement with those found in the present study, benign colon polyps showed a significantly lower *Km*ADP [18], suggesting an early energy metabolism switch, mediated by changes in the energy transfer pathway during CRC development.

Moreover, the higher Vmax values and *Vmax*/*Km*ADP ratios in CRC suggested an increased OxPhos capacity and efficiency. These data confirmed the earlier findings [18], suggesting that high OxPhos *Vmax* values (and *Vmax*/*Km*ADP ratios) could be a marker for a more aggressive disease. There was one recent CRC patient among the group of alive patients who showed a high *Vmax* value of 4.21, which was left out of the calculations as an outlier. However, it could be interesting to follow this patient's disease progression to validate the hypothesis of high *Vmax* being a prognostic marker.

There are several possible explanations for why the lethal Stage III‐IV group did not show a significant difference in *KmADP*. Firstly, the group contained only two patients; secondly, advanced stage tumours have grown into nearby tissues including muscle tissue in the colon wall, meaning that these samples may include some muscle tissue that is characterised by a high *KmADP*. As described before, a lower *KmADP* value could indicate a shift to glycolytic metabolism. In turn, a high *Vmax* may derive from an advanced vascularisation in more aggressive malignant tumours and from an enhanced energy demand for metastatic processes. Interestingly, our previous work has shown a similar high *Vmax*—low *KmADP* metabolic profile in colon polyps, which are benign growths in the colon mucosa [18].

Although therapeutic strategies for Stage I, III and IV CRC are well established, the treatment approach for Stage II remains a subject of debate. Stage II is recognised as a heterogeneous category, with prognoses varying significantly. The search for more effective therapeutic strategies has led to the exploration of various biomarkers [56]. Our results suggested the surge of a critical metabolic anomality within Stage II CRC, perhaps reflecting changes in energetic needs and epithelial‐mesenchymal transition onset that warrants further investigation into the specifics of its energy metabolism and energy‐dependent metastasis events.

Furthermore, the data suggested that distinct energy metabolism profiles may emerge for the various colorectal tumour stages and their progression from one stage to another. Although the metabolic differences among CRC Stages I, II and III might seem inherently minimal, this may have derived from the relatively small sample size examined. Our study did not include cases of high‐grade metastatic CRC, which previous research has shown to exhibit a notable increase in EC by approximately 26%, whereas CRC Stages I and III have shown negligible changes in EC [57], like the ones described here.

The lack of significant differences in the kinetic parameters of the ADP‐stimulated respiration rate (Vmax and Km) across CRC stages observed in this study may be explained by the high heterogeneity of the samples and limited patient grouping. Specifically, the current study used treatment‐naïve patients that are typically categorised as Stage I or II, while Stages III and IV are rarely surgically treated without prior chemotherapy or radiotherapy. These factors may have contributed to masking the subtle variations in EC that could otherwise indicate stage‐specific metabolic alterations. Hence, a larger and more comprehensively designed cohort may be essential to further refine this approach and accurately identify differences in EC associated with CRC progression. Nevertheless, when the *Vmax/Km* ratio or catalytic efficiency is considered (*cf*. Figure 6C), or the EC values of Stage II are compared to those of control tissue samples (cf. Figure 6D), statistical significance emerged. Therefore, it seems the *Vmax/Km* ratio is a more sensitive parameter which might be able to help distinguish differences in the energy metabolism status of the different CRC stages. Let us recall that the catalytic efficiency comprises both catalytic capacity and substrate affinity, the two main kinetic parameters of an enzyme or metabolic pathway.

It is noted that EC and ATP/ADP ratio values were obtained from fully ADP‐stimulated OxPhos samples. Then, to get a closer physiological view of colorectal cancer, a similar analysis should be undertaken for tumour samples under glycolysis and under both OxPhos and glycolysis to resemble actual microenvironmental changes undergone by CRC.

From the results described, it is clear that energy metabolism undergoes reprogramming throughout CRC development. We hypothesise that benign colon polyps increase glycolytic activity to support the increased proliferation by mainly supplying anabolic precursors such as Glc6P, Fru6P, DHAP, 3PG and Pyr and helping with the elevated energy demand under hypoxic conditions [8]. As the cell number increases and the tumour grows in size, the cells become deprived of oxygen and nutrients; consequently, new blood vessels are generated in the tissue to restore the supply.

Hanahan and Weinberg have proposed induced angiogenesis as one of the hallmarks of cancer [58]. While angiogenesis is usually dormant in adults, it is reinduced in early stages of tumorigenesis to further support cell growth and proliferation. As the cancer progresses even further, cancer tissue will become more and more heterogeneous. Some cells will persist in hypoxic conditions and rely predominantly on glycolysis, while others, benefiting from sufficient oxygen availability, utilise lactate produced by the former cells as a substrate for ATP production via OxPhos [8, 59, 60]. Thus, it has become apparent that there is metabolic heterogeneity and plasticity within solid tumours [10, 61, 62, 63] and there is growing evidence of hybrid energy metabolism phenotypes in several cancer cell lines and tumours [7, 8, 9, 10, 62]. Depending on the microenvironment, glycolysis or OxPhos or both may become predominant for energy supply [7, 8, 9, 59, 60, 63].

## 5 Conclusion

The data of the present study showed that colorectal human tumours display altered expression, protein contents and enzyme activities of their energy metabolism, including the energy transfer pathway. The contents and activities of some glycolytic proteins, the changes in the energy charge values, enhanced OxPhos flux and catalytic efficiency suggested that colorectal tumours have highly active glycolysis and OxPhos; therefore, they seem to display a hybrid energy metabolism phenotype. Such a hybrid metabolic phenotype in CRC indicates drug targeting of both pathways, either with multi‐site drugs or a combination of drugs, to achieve successful treatment schemes. Drugs preferentially or specifically targeting cancer mitochondria and glycolysis, particularly lipophilic cationic, vitamin E analogues or cholesterol‐binding molecules, should be tested [7, 8, 11].

Moreover, the present study underscores the complex nature of cancer, advocating for an integral approach that employs both cancer cell cultures and tumour clinical material. This comprehensive approach has unveiled instances previously unseen, which might be challenging or even impossible to discern through more conventional or isolated research strategies. The present findings highlight the importance of combining diverse experimental models to fully understand the multifaceted biological behaviours of cancer.

## Author Contributions

**Leenu Reinsalu:** conceptualization (equal), formal analysis (equal), investigation (equal), methodology (equal), visualization (equal), writing – original draft (equal). **Sten Miller:** conceptualization (equal), formal analysis (equal), investigation (equal), methodology (equal), visualization (equal), writing – original draft (equal). **Giuseppe Leonardo Auditano:** formal analysis (equal), investigation (supporting), methodology (supporting), writing – review and editing (equal). **Marju Puurand:** formal analysis (supporting), investigation (supporting). **Rafael Moreno‐Sanchez:** conceptualization (equal), funding acquisition (equal), writing – review and editing (equal). **Emma Saavedra:** writing – review and editing (equal). **Vahur Valvere:** methodology (equal), resources (equal). **Tuuli Käämbre:** conceptualization (equal), funding acquisition (equal), supervision (equal), writing – review and editing (equal).

## Ethics Statement

The studies involving human participants were reviewed and approved by the Research Ethics Committee of the National Institute for Health Development in Estonia (decision numbers KK557 and KK558) and followed the Helsinki Declaration and Convention of the Council of Europe on Human Rights and Biomedicine. All participants were informed about the study and provided their written informed consent to participate in the study.

## Conflicts of Interest

The authors declare no conflicts of interest.

## Data Availability

The data that support the findings of this study are available from the corresponding author upon request.

## References

[1] World Health Organization , “Cancer,” (2022).

[2] Centers for Disease Control and Prevention , “U.S. Cancer Statistics Colorectal Cancer Stat Bite,” (2023).

[3] R. Udo , K. Katsumata , H. Kuwabara , et al., “Urinary Charged Metabolite Profiling of Colorectal Cancer Using Capillary Electrophoresis‐Mass Spectrometry,” Scientific Reports 10, no. 1 (2020): 21057, 10.1038/s41598-020-78038-2. 33273632 PMC7713069

[4] F. di Cesare , A. Vignoli , C. Luchinat , L. Tenori , and E. Saccenti , “Exploration of Blood Metabolite Signatures of Colorectal Cancer and Polyposis Through Integrated Statistical and Network Analysis,” Metabolites 13 (2023): 296, 10.3390/metabo13020296. 36837915 PMC9965766

[5] D. Hanahan and R. A. Weinberg , “Hallmarks of Cancer: The Next Generation,” Cell 144, no. 5 (2011): 646–674, 10.1016/j.cell.2011.02.013. 21376230

[6] O. Warburg , “On the Origin of Cancer Cells,” Science 123, no. 3191 (1956): 309–314, 10.1126/science.123.3191.309. 13298683

[7] R. Moreno‐Sanchez , S. Rodriguez‐Enriquez , A. Marin‐Hernandez , and E. Saavedra , “Energy Metabolism in Tumor Cells,” FEBS Journal 274, no. 6 (2007): 1393–1418, 10.1111/j.1742-4658.2007.05686.x. 17302740

[8] R. Moreno‐Sanchez , A. Marin‐Hernandez , E. Saavedra , J. P. Pardo , S. J. Ralph , and S. Rodriguez , “Enriquez: Who Controls the ATP Supply in Cancer Cells? Biochemistry Lessons to Understand Cancer Energy Metabolism,” International Journal of Biochemistry & Cell Biology 50 (2014): 10–23, 10.1016/j.biocel.2014.01.025. 24513530

[9] R. Moreno‐Sánchez , D. X. Robledo‐Cadena , S. C. Pacheco‐Velázquez , J. L. Vargas Navarro , J. A. Padilla‐Flores , and S. Rodríguez‐Enríquez , “Estimation of Energy Pathway Fluxes in Cancer Cells—Beyond the Warburg Effect,” Archives of Biochemistry and Biophysics 739 (2023): 109559, 10.1016/j.abb.2023.109559. 36906097

[10] L. Reinsalu , M. Puurand , V. Chekulayev , et al., “Energy Metabolic Plasticity of Colorectal Cancer Cells as a Determinant of Tumor Growth and Metastasis,” Frontiers in Oncology 11 (2021): 698951, 10.3389/fonc.2021.698951. 34381722 PMC8351413

[11] R. Moreno‐Sánchez , E. Saavedra , J. C. Gallardo‐Pérez , F. D. Rumjanek , and S. Rodríguez‐Enríquez , “Understanding the Cancer Cell Phenotype Beyond the Limitations of Current Omics Analyses,” FEBS Journal 283, no. 1 (2016): 54–73, 10.1111/febs.13535. 26417966

[12] M. V. Golikov , V. T. Valuev‐Elliston , O. A. Smirnova , and A. V. Ivanov , “Physiological Media in Studies of Cell Metabolism,” Molecular Biology 56, no. 5 (2022): 629–637, 10.1134/s0026893322050077. 36217338 PMC9534458

[13] A. Untergasser , RNAprep – Trizol Combined With Columns, vol. 2023 ( *Untergasser*'*s Lab* , 2008).

[14] E. K. Seppet , T. Kaambre , P. Sikk , et al., “Functional Complexes of Mitochondria With ca, MgATPases of Myofibrils and Sarcoplasmic Reticulum in Muscle Cells,” Biochimica et Biophysica Acta 1504, no. 2–3 (2001): 379–395. 11245802 10.1016/s0005-2728(00)00269-3

[15] P.‐B. Ancey , C. Contat , G. Boivin , et al., “GLUT1 Expression in Tumor‐Associated Neutrophils Promotes Lung Cancer Growth and Resistance to Radiotherapy,” Cancer Research 81, no. 9 (2021): 2345–2357, 10.1158/0008-5472.can-20-2870. 33753374 PMC8137580

[16] P.‐B. Ancey , C. Contat , and E. Meylan , “Glucose Transporters in Cancer – From Tumor Cells to the Tumor Microenvironment,” FEBS Journal 285, no. 16 (2018): 2926–2943, 10.1111/febs.14577. 29893496

[17] H. Xiao , J. Wang , W. Yan , et al., “GLUT1 Regulates Cell Glycolysis and Proliferation in Prostate Cancer,” Prostate 78, no. 2 (2018): 86–94, 10.1002/pros.23448. 29105798

[18] E. Rebane‐Klemm , L. Reinsalu , M. Puurand , et al., “Colorectal Polyps Increase the Glycolytic Activity,” Frontiers in Oncology 13 (2023): e1171887, 10.3389/fonc.2023.1171887. PMC10277630 37342183

[19] A. Kaldma , A. Klepinin , V. Chekulayev , et al., “An In Situ Study of Bioenergetic Properties of Human Colorectal Cancer: The Regulation of Mitochondrial Respiration and Distribution of Flux Control Among the Components of ATP Synthasome,” International Journal of Biochemistry & Cell Biology 55 (2014): 171–186, 10.1016/j.biocel.2014.09.004. 25218857

[20] J. Joseph , A. Cardesa , and J. Carreras , “Creatine Kinase Activity and Isoenzymes in Lung, Colon and Liver Carcinomas,” British Journal of Cancer 76, no. 5 (1997): 600–605. 9303358 10.1038/bjc.1997.432 PMC2228007

[21] C. Panayiotou , N. Solaroli , and A. Karlsson , “The Many Isoforms of Human Adenylate Kinases,” International Journal of Biochemistry & Cell Biology 49 (2014): 75–83, 10.1016/j.biocel.2014.01.014. 24495878

[22] C.‐C. Hsu , D. Peng , Z. Cai , and H.‐K. Lin , “AMPK Signaling and Its Targeting in Cancer Progression and Treatment,” Seminars in Cancer Biology 85 (2022): 52–68, 10.1016/j.semcancer.2021.04.006. 33862221 PMC9768867

[23] A. Koit , I. Shevchuk , L. Ounpuu , et al., “Mitochondrial Respiration in Human Colorectal and Breast Cancer Clinical Material Is Regulated Differently,” Oxidative Medicine and Cellular Longevity 2017 (2017): 1372640, 10.1155/2017/1372640. 28781720 PMC5525093

[24] A. Marín‐Hernández , J. C. Gallardo‐Pérez , S. Rodríguez‐Enríquez , R. Encalada , R. Moreno‐Sánchez , and E. Saavedra , “Modeling Cancer Glycolysis,” BBA–Bioenergetics 1807 (2011): 755–767. 21110941 10.1016/j.bbabio.2010.11.006

[25] M. Katagiri , H. Karasawa , K. Takagi , et al., “Hexokinase 2 in Colorectal Cancer: A Potent Prognostic Factor Associated With Glycolysis, Proliferation and Migration,” Histology and Histopathology 32, no. 4 (2017): 351–360, 10.14670/HH-11-799. 27363977

[26] P. L. Pedersen , S. Mathupala , A. Rempel , J. F. Geschwind , and Y. H. Ko , “Mitochondrial Bound Type II Hexokinase: A Key Player in the Growth and Survival of Many Cancers and an Ideal Prospect for Therapeutic Intervention,” Biochimica et Biophysica Acta (BBA) Bioenergetics 1555, no. 1 (2002): 14–20, 10.1016/S0005-2728(02)00248-7. 12206885

[27] L. Ounpuu , L. Truu , I. Shevchuk , et al., “Comparative Analysis of the Bioenergetics of Human Adenocarcinoma Caco‐2 Cell Line and Postoperative Tissue Samples From Colorectal Cancer Patients,” Biochemistry and Cell Biology 96, no. 6 (2018): 808–817, 10.1139/bcb-2018-0076. 30058357

[28] Z. Xie , H. Zhou , L. Wang , and Y. Wu , “The Significance of the Preoperative Lactate Dehydrogenase/Albumin Ratio in the Prognosis of Colon Cancer: A Retrospective Study,” PeerJ 10 (2022): e13091, 10.7717/peerj.13091. 35295561 PMC8919845

[29] A. Casadei‐Gardini , E. Scarpi , P. Ulivi , et al., “Prognostic Role of a New Inflammatory Index With Neutrophil‐To‐Lymphocyte Ratio and Lactate Dehydrogenase (CII: Colon Inflammatory Index) in Patients With Metastatic Colorectal Cancer: Results From the Randomized Italian Trial in Advanced Colorectal Cancer (ITACa) Study,” Cancer Management and Research 11 (2019): 4357–4369, 10.2147/CMAR.S198651. 31191000 PMC6522652

[30] M. Manerba , L. di Ianni , M. Govoni , M. Roberti , M. Recanatini , and G. di Stefano , “Lactate Dehydrogenase Inhibitors Can Reverse Inflammation Induced Changes in Colon Cancer Cells,” European Journal of Pharmaceutical Sciences 96 (2017): 37–44, 10.1016/j.ejps.2016.09.014. 27622920

[31] R. Moreno‐Sanchez , J. L. Vargas‐Navarro , J. A. Padilla‐Flores , et al., “Energy Metabolism Behavior and Response to Microenvironmental Factors of the Experimental Cancer Cell Models Differ From That of Actual Human Tumors,” Mini Reviews in Medicinal Chemistry 25, no. 4 (2024), 10.2174/0113895575322436240924101642 Epub ahead of print. 39411957

[32] V. L. Payen , E. Mina , V. F. van Hee , P. E. Porporato , and P. Sonveaux , “Monocarboxylate Transporters in Cancer,” Molecular Metabolism 33 (2020): 48–66, 10.1016/j.molmet.2019.07.006. 31395464 PMC7056923

[33] S. Rodríguez‐Enríquez , D. X. Robledo‐Cadena , J. C. Gallardo‐Pérez , et al., “Acetate Promotes a Differential Energy Metabolic Response in Human HCT 116 and COLO 205 Colon Cancer Cells Impacting Cancer Cell Growth and Invasiveness,” Frontiers in Oncology 11 (2021): e697408, 10.3389/fonc.2021.697408. PMC8370060 34414111

[34] R. Hussien and G. A. Brooks , “Mitochondrial and Plasma Membrane Lactate Transporter and Lactate Dehydrogenase Isoform Expression in Breast Cancer Cell Lines,” Physiological Genomics 43, no. 5 (2011): 255–264, 10.1152/physiolgenomics.00177.2010. 21177384 PMC3068517

[35] V. Chekulayev , K. Mado , I. Shevchuk , et al., “Metabolic Remodeling in Human Colorectal Cancer and Surrounding Tissues: Alterations in Regulation of Mitochondrial Respiration and Metabolic Fluxes,” Biochemistry and Biophysics Reports 4 (2015): 111–125, 10.1016/j.bbrep.2015.08.020. 29124194 PMC5668899

[36] U. Schlattner , M. Tokarska‐Schlattner , and T. Wallimann , “Mitochondrial Creatine Kinase in Human Health and Disease,” Biochimica et Biophysica Acta 1762, no. 2 (2006): 164–180, 10.1016/j.bbadis.2005.09.004. 16236486

[37] P. Dzeja and A. Terzic , “Adenylate Kinase and AMP Signaling Networks: Metabolic Monitoring, Signal Communication and Body Energy Sensing,” International Journal of Molecular Sciences 10, no. 4 (2009): 1729–1772, 10.3390/ijms10041729. 19468337 PMC2680645

[38] D. M. Bai , J. F. Zhang , T. T. Li , et al., “The ATPase hCINAP Regulates 18S rRNA Processing and Is Essential for Embryogenesis and Tumour Growth,” Nature Communications 7 (2016): 12310, 10.1038/ncomms12310. PMC4974663 27477389

[39] Y.‐B. Yan , “Creatine Kinase in Cell Cycle Regulation and Cancer,” Amino Acids 48, no. 8 (2016): 1775–1784, 10.1007/s00726-016-2217-0. 27020776

[40] S. M. Mooney , K. Rajagopalan , B. H. Williams , et al., “Creatine Kinase Brain Overexpression Protects Colorectal Cells From Various Metabolic and Non‐Metabolic Stresses,” Journal of Cellular Biochemistry 112, no. 4 (2011): 1066–1075, 10.1002/jcb.23020. 21308735 PMC4380256

[41] A. Klepinin , S. Zhang , L. Klepinina , et al., “Adenylate Kinase and Metabolic Signaling in Cancer Cells,” Frontiers in Oncology 10 (2020): 660, 10.3389/fonc.2020.00660. 32509571 PMC7248387

[42] J. Liang and G. B. Mills , “AMPK: A Contextual Oncogene or Tumor Suppressor?,” Cancer Research 73, no. 10 (2013): 2929–2935, 10.1158/0008-5472.CAN-12-3876. 23644529 PMC3725287

[43] W. Wang and K. L. Guan , “AMP‐Activated Protein Kinase and Cancer,” Acta Physiologica 196, no. 1 (2009): 55–63, 10.1111/j.1748-1716.2009.01980.x. 19243571

[44] B. Faubert , G. Boily , S. Izreig , et al., “AMPK Is a Negative Regulator of the Warburg Effect and Suppresses Tumor Growth In Vivo,” Cell Metabolism 17, no. 1 (2013): 113–124. 23274086 10.1016/j.cmet.2012.12.001 PMC3545102

[45] G. Rehman , A. Shehzad , A. L. Khan , and M. Hamayun , “Role of AMP‐Activated Protein Kinase in Cancer Therapy,” Archiv der Pharmazie 347, no. 7 (2014): 457–468, 10.1002/ardp.201300402. 24677093

[46] D. G. Hardie , F. A. Ross , and S. A. Hawley , “AMP‐Activated Protein Kinase: A Target for Drugs Both Ancient and Modern,” Chemistry & Biology 19, no. 10 (2012): 1222–1236, 10.1016/j.chembiol.2012.08.019. 23102217 PMC5722193

[47] H. U. Park , S. Suy , M. Danner , et al., “AMP‐Activated Protein Kinase Promotes Human Prostate Cancer Cell Growth and Survival,” Molecular Cancer Therapeutics 8, no. 4 (2009): 733–741, 10.1158/1535-7163.MCT-08-0631. 19372545 PMC2775041

[48] A. S. Khan and D. E. Frigo , “Regulation, Role and Therapeutic Targeting of AMPK in Prostate Cancer,” Nature Reviews. Urology 14, no. 3 (2017): 164–180, 10.1038/nrurol.2016.272. 28169991 PMC5672799

[49] X. Huang , X. Li , X. Xie , et al., “High Expressions of LDHA and AMPK as Prognostic Biomarkers for Breast Cancer,” Breast 30 (2016): 39–46, 10.1016/j.breast.2016.08.014. 27598996

[50] L. Collavin , D. Lazarevič , R. Utrera , S. Marzinotto , M. Monte , and C. Schneider , “Wt p53 Dependent Expression of a Membrane‐Associated Isoform of Adenylate Kinase,” Oncogene 18, no. 43 (1999): 5879–5888, 10.1038/sj.onc.1202970. 10557075

[51] D. E. Atkinson and G. M. Walton , “Adenosine Triphosphate Conservation in Metabolic Regulation: Rat Liver Citrate Cleavage Enzyme,” Journal of Biological Chemistry 242, no. 13 (1967): 3239–3241, 10.1016/S0021-9258(18)95956-9. 6027798

[52] L. Iommarini , A. Ghelli , G. Gasparre , and A. M. Porcelli , “Mitochondrial Metabolism and Energy Sensing in Tumor Progression,” Biochimica et Biophysica Acta (BBA) Bioenergetics 1858, no. 8 (2017): 582–590, 10.1016/j.bbabio.2017.02.006. 28213331

[53] I. M. de la Fuente , J. M. Cortés , E. Valero , et al., “On the Dynamics of the Adenylate Energy System: Homeorhesis vs Homeostasis,” PLoS One 9, no. 10 (2014): e108676, 10.1371/journal.pone.0108676. 25303477 PMC4193753

[54] Frederick Jay Passman , Jordan Schmidt , and Russell P Lewis , “The Relationship Between Microbial Community Vitality and ATP Bioburden in Bottom Waters Under Fuel Microcosms,” Access Microbiology 5, no. 4 (2023): acmi000411, 10.1099/acmi.0.000411. 37223058 PMC10202405

[55] A. V. Kuznetsov , T. Tiivel , P. Sikk , et al., “Striking Differences Between the Kinetics of Regulation of Respiration by ADP in Slow‐Twitch and Fast‐Twitch Muscles In Vivo,” European Journal of Biochemistry 241, no. 3 (1996): 909–915, 10.1111/j.1432-1033.1996.00909.x. 8944782

[56] P. Parent , R. Cohen , E. Rassy , et al., “A Comprehensive Overview of Promising Biomarkers in Stage II Colorectal Cancer,” Cancer Treatment Reviews 88 (2020): e102059, 10.1016/j.ctrv.2020.102059. 32622273

[57] Z. Teo , M. K. Sng , J. S. K. Chan , et al., “Elevation of Adenylate Energy Charge by Angiopoietin‐Like 4 Enhances Epithelial–Mesenchymal Transition by Inducing 14‐3‐3γ Expression,” Oncogene 36, no. 46 (2017): 6408–6419, 10.1038/onc.2017.244. 28745316 PMC5701092

[58] D. Hanahan and R. A. Weinberg , “The Hallmarks of Cancer,” Cell 100, no. 1 (2000): 57–70, 10.1016/s0092-8674(00)81683-9. 10647931

[59] S. J. Ralph , S. Rodríguez‐Enríquez , J. Neuzil , E. Saavedra , and R. Moreno‐Sánchez , “The Causes of Cancer Revisited: ‘Mitochondrial Malignancy’ and ROS‐Induced Oncogenic Transformation – Why Mitochondria Are Targets for Cancer Therapy,” Molecular Aspects of Medicine 31, no. 2 (2010): 145–170. 20206201 10.1016/j.mam.2010.02.008

[60] R. Moreno‐Sanchez , E. Saavedra , S. Rodriguez‐Enriquez , J. C. Gallardo‐Perez , H. Quezada , and H. V. Westerhoff , “Metabolic Control Analysis Indicates a Change of Strategy in the Treatment of Cancer,” Mitochondrion 10, no. 6 (2010): 626–639, 10.1016/j.mito.2010.06.002. 20599628

[61] C. T. Hensley , B. Faubert , Q. Yuan , et al., “Metabolic Heterogeneity in Human Lung Tumors,” Cell 164, no. 4 (2016): 681–694, 10.1016/j.cell.2015.12.034. 26853473 PMC4752889

[62] D. Jia , J. H. Park , K. H. Jung , H. Levine , and B. A. Kaipparettu , “Elucidating the Metabolic Plasticity of Cancer: Mitochondrial Reprogramming and Hybrid Metabolic States,” Cells 7, no. 3 (2018): 21, 10.3390/cells7030021. 29534029 PMC5870353

[63] S. Rodríguez‐Enríquez , T. Kaambre , and R. Moreno‐Sánchez , “Editorial: Metabolic Plasticity of Cancer,” Frontiers in Oncology 10 (2020): 599723, 10.3389/fonc.2020.599723. 33194768 PMC7645106

